# Identification and Functional Characterization of Genes Encoding Omega-3 Polyunsaturated Fatty Acid Biosynthetic Activities from Unicellular Microalgae

**DOI:** 10.3390/md11125116

**Published:** 2013-12-16

**Authors:** Royah Vaezi, Johnathan A. Napier, Olga Sayanova

**Affiliations:** Department of Biological Chemistry and Crop Protection, Rothamsted Research, Harpenden, Herts AL5 2JQ, UK; E-Mails: royah.vaezi@rothamsted.ac.uk (R.V.); johnathan.napier@rothamsted.ac.uk (J.A.N.)

**Keywords:** microalgae, omega-3 long chain polyunsaturated fatty acid, desaturases, elongases

## Abstract

In order to identify novel genes encoding enzymes involved in the biosynthesis of nutritionally important omega-3 long chain polyunsaturated fatty acids, a database search was carried out in the genomes of the unicellular photoautotrophic green alga *Ostreococcus* RCC809 and cold-water diatom *Fragilariopsis cylindrus*. The search led to the identification of two putative “front-end” desaturases (Δ6 and Δ4) from *Ostreococcus* RCC809 and one Δ6-elongase from *F. cylindrus.* Heterologous expression of putative open reading frames (ORFs) in yeast revealed that the encoded enzyme activities efficiently convert their respective substrates: 54.1% conversion of α-linolenic acid for Δ6-desaturase, 15.1% conversion of 22:5*n*-3 for Δ4-desaturase and 38.1% conversion of γ-linolenic acid for Δ6-elongase. The Δ6-desaturase from *Ostreococcus* RCC809 displays a very strong substrate preference resulting in the predominant synthesis of stearidonic acid (C18:4Δ^6,9,12,15^). These data confirm the functional characterization of omega-3 long chain polyunsaturated fatty acid biosynthetic genes from these two species which have until now not been investigated for such activities. The identification of these new genes will also serve to expand the repertoire of activities available for metabolically engineering the omega-3 trait in heterologous hosts as well as providing better insights into the synthesis of eicosapentaenoic acid (EPA) and docosahexaenoic acid (DHA) in marine microalgae.

## 1. Introduction

It is now well accepted that omega-3 long chain polyunsaturated fatty acids (LC-PUFAs), especially eicosapentaenoic acid (EPA; 20:5Δ5,8,11,14,17) and docosahexaenoic acid (DHA; 22:6Δ4,7,10,13,16,19) are vital for human health and nutrition and play a crucial role in preventing cardiovascular diseases and associated precursor conditions such as metabolic syndrome and obesity [1,2]. Currently, oily marine fish is the major dietary source of these fatty acids; however, considering growing pressure on global fish stocks and pollution of the marine environment there is an urgent need for an alternative cost-effective solution for large-scale production of LC-PUFAs.

**Figure 1:**
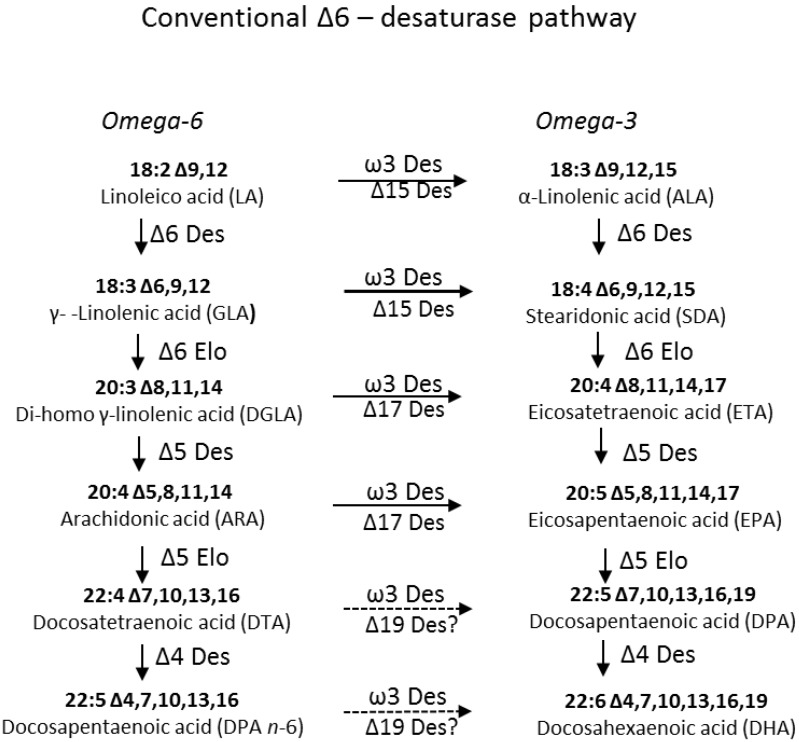
Pathway for the biosynthesis of long chain polyunsaturated fatty acids (LC-PUFAs) in microalgae.

In recent years, the feasibility of using higher plants for the production of omega-3 LC-PUFAs has been explored and considerable progress has been made in effective production of EPA and DHA in oilseeds [3,4,5]. A variety of strategies have been used to introduce (via genetic engineering) the omega-3 LC-PUFA metabolic pathways in oil crops, mainly by expressing desaturase and elongase genes involved in different biosynthetic routes for EPA and DHA accumulation [6]. Marine algae are the primary producers of omega-3 LC-PUFAs and therefore represent the logical source for the identification of genes encoding the enzymes required for the synthesis of EPA and DHA. Most omega-3 LC-PUFA-synthesising marine organisms utilize the so-called Δ6-desaturase “conventional” aerobic pathway which relies on a consecutive series of altering desaturation and elongation steps to convert α-linolenic acid (ALA; 18:3Δ9,12,15) to EPA and DHA (Figure 1). The first step in this pathway is the Δ6-desaturation of both linoleic acid (LA; 18:2Δ9,12) and ALA, resulting in the synthesis of γ-linolenic acid (GLA; 18:3Δ6,9,12) and stearidonic acid (SDA; 18:4Δ6,9,12,15), respectively. This step is followed by a Δ6-specific C2 elongation, yielding di-homo γ-linolenic acid (DGLA; 20:3Δ8,11,14) and eicosatetraenoic acid (ETA; 20:4Δ8,11,14,17). Finally, these LC-PUFAs are desaturated by a Δ5-desaturase to generate arachidonic acid (ARA; 20:4Δ5,8,11,14) and EPA, respectively. In DHA-accumulating microorganisms, the pathway involves C2 elongation of EPA to docosapentaenoic acid (DPA; 22:5 Δ7,10,13,16,19) by a specific Δ5-elongase which is then desaturated by a Δ4-specific desaturase to yield DHA. Although most enzymes involved in this pathway show limited discrimination between n-3 and n-6 acyl-substrates, the predominant presence of *n*-3 LC-PUFAs in most marine microorganisms indicates the likely presence of ω-3-desaturases which convert omega-6 to omega-3. However, some examples of Δ6-desaturases cloned from microalgae show a preference for ω-3 substrates (as was reported for higher plant Δ6-desaturases from *Primula* spp and *Echium* [7,8,9]. An ω-3 substrate preference has previously been described for enzymes from *Mantoniella squamata* [10], *Micromonas pusilla* [11] and *Ostreococcus lucimarinus* [12].

In this present study, we examined the fatty acid profile of two marine microalgae, the unicellular photoautotrophic green alga *Ostreococcus* RCC809 and the cold-water diatom *Fragilariopsis cylindrus*, both of which have previously only been subject to limited investigation as to the nature of their synthesis and accumulation of omega-3 LC-PUFAs. We also used on-going genomic sequencing projects for these two organisms to identify and functionally characterize three examples of genes involved in the biosynthesis of EPA and DHA. Interestingly, one particular enzyme (Δ6-desaturase from *Ostreococcus* RCC809) showed a strong preference of ω-3 substrates against ω-6.

## 2. Results and Discussion

### 2.1. Identification and Functional Characterization of *Ostreococcus* RCC809 Genes for “Front-End” Desaturases

#### 2.1.1. Fatty Acid Composition of *Ostreococcus* RCC809

**Figure 2:**
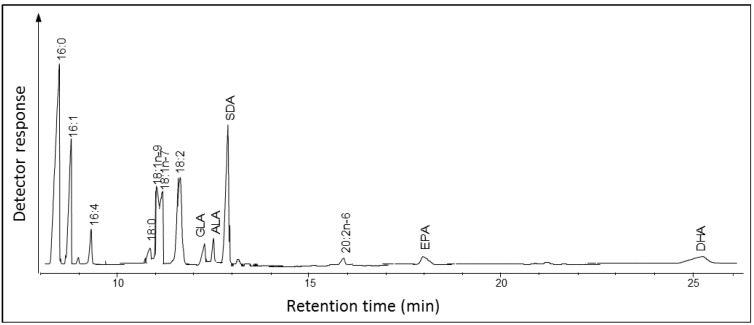
Total fatty acid methyl esters of *Ostreococcus* RCC809*.* Fatty acid methyl esters (FAMEs) were prepared and analyzed by gas chromatography coupled with a flame ionization detector (GC-FID) as described in “Experimental Section”, with peaks being identified by co-migration against known standards. The identity of major peaks is shown.

*Ostreococcus* RCC809 is the smallest known free-leaving marine picophytoeukaryote belonging to the “low-light” adapted ecotype of *Prasinophyceae* [13]*.* It is closely related to the two “high-light” adapted ecotypes of the genus *Ostreococcus*, *O. tauri* and *O. lucimarinus*, that have the ability to synthesize EPA and DHA via a series of alternating desaturation and elongation steps [12,13,14,15,16]. To explore the LC-PUFA pathway operating in *Ostreococcus* RCC809 we analyzed by GC-FID and GC-MS the fatty acid methyl esters (FAMEs) of total lipids from *Ostreococcus* RCC809 cultures growing at stationary phase. This analysis revealed the presence of several fatty acids belonging to the *n*-3 PUFA pathway (Figure 2). The most abundant fatty acid was 16:0 (20.1% of total fatty acids, TFA) followed by SDA (19.1% of TFA) and 16:1 (18.1% of TFA) (Supplementary Table S1). Compared to *O. tauri*, which contains on average 12% of DHA [13], the amount of this fatty acid in *Ostreococcus* RCC809 is rather low (1.83%); similarly, the levels of EPA are very low*.* The fatty acid content of *Ostreococcus* RCC809 is comparable to what has been found for *O. lucimarinus*, containing high levels of SDA (15%) and small amount of EPA and DHA [17]. Thus, the predominant omega-3 PUFA in *Ostreococcus* RCC809 is the Δ6-desaturated C18 fatty acid SDA, as opposed to either EPA or DHA.

#### 2.1.2. Identification and Functional Characterization in Yeast of a Putative Δ6-Desaturase from *Ostreococcus* RCC809

The genome of the green alga *Ostreococcus* RCC809 has been sequenced by US DOE Joint Genome Institute (JGI) and the predicted gene models are available for inspection and query [18]. We queried the predicted gene models via BLAST using previously characterized *N*-terminal cytochrome b5-fusion desaturases as query sequences. This analysis revealed the presence of several genes coding for putative omega-3 LC-PUFA desaturases. The top-scoring predicted open reading frames (ORFs) are listed in Table 1. Interestingly, no obvious Δ5-desaturase was identified by our searches, as might be expected for an organism that synthesizes EPA (Figure 1, Supplementary Table S1). The most obvious explanation for this absence is that the sequence of the *Ostreococcus* RCC809 genome is not yet complete and/or the genomic structure of the Δ5-desaturase is such that it has evaded the gene-prediction algorithms used to identify ORFs. The enzymatic activities of the two identified candidate desaturases (Table 1) were investigated by heterologous expression in *Saccharomyces cerevisiae*, and the deduced open reading frames were used as templates to chemically synthesize codon-optimized nucleotide sequences for expression in diatoms (based on the subsequent requirement to express the algal sequences in transgenic *Phaeodactylum tricornutum* and our observation that such diatom codon usage was readily accepted by *S. cerevisiae*). These synthetic coding sequences were cloned as *KpnI-SacI* fragments behind the galactose–inducible GAL1 promoter of the yeast expression vector pYES2 (Invitrogen, Carlsbad, CA, USA) and expressed in yeast in the presence of potential fatty acid substrates as previously described [7]. Total fatty acid methyl esters (FAMEs) from transgenic yeast were analyzed by GC-FID and the identity of novel peaks confirmed by GC-MS and co-migration with authentic standards.

**Table 1:** LC-PUFA biosynthetic genes cloned from *Ostreococcus* RCC809.

Species	Protein ID	Amino Acids	Closest Match on Genbank, % Identity	Designation	Defined Function
*Ostreococcus* RCC809	59992	461	Δ6-desaturase from *O.lucimarinus* (82%) Accession number: DAA34893.1	**Ost809D6**	C18 Δ6-desaturase
*Ostreococcus* RCC809	40461	459	Δ4-desaturase from *O. lucimarinus* (85%) Accession number: XP_001415743.1	**Ost809D4**	C22 Δ4-desaturase

Blast analysis using as a query the amino acid sequence of protein 59992 showed that the protein had high homology to previously reported acyl-CoA Δ6-desaturases from microalgae (Supplementary Figure S1). The protein from *Ostreococcus* RCC809 was most similar to the Δ6-desaturases from its closest relatives, *O. lucimarinus* and *O. tauri* which had an identity of 82% and 75%, respectively.

**Figure 3:**
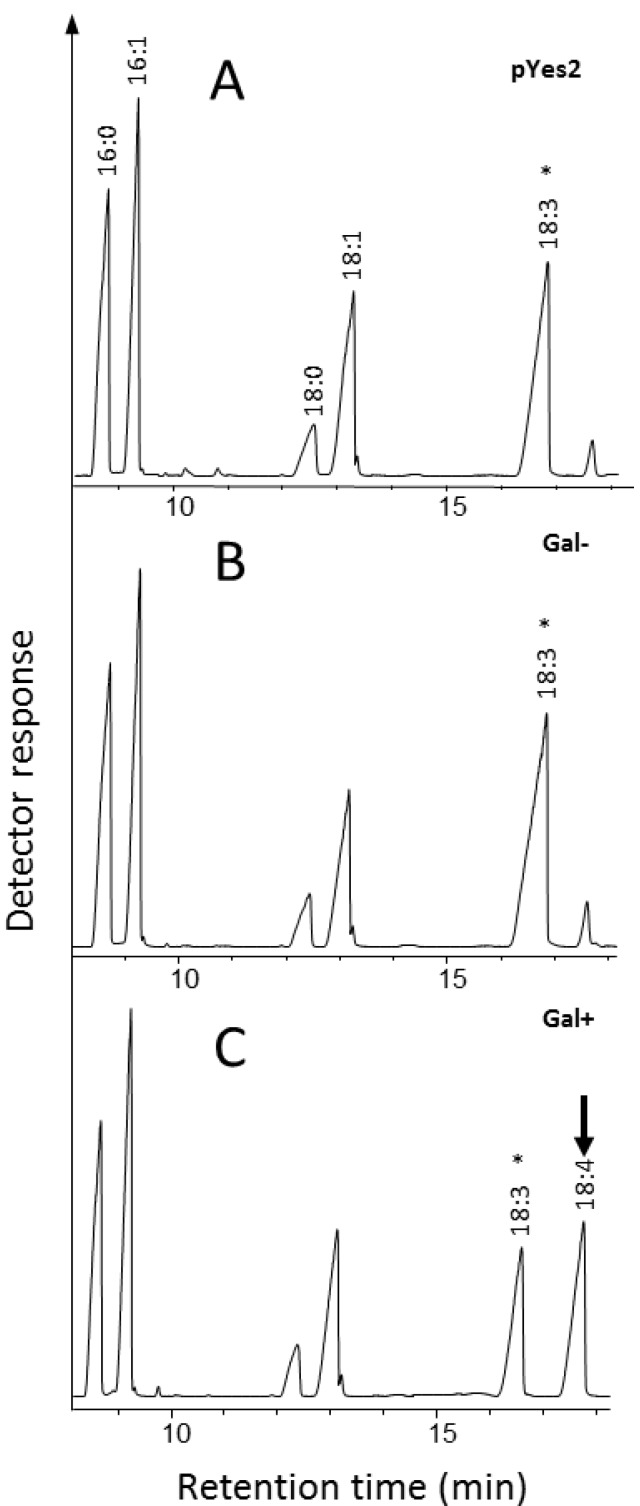
Functional characterization of *Ostreococcus* RCC809 C18 Δ6-desaturase. A synthetic gene encoding *Ostreococcus* RCC809 Protein 59992 was expressed in *S. cerevisiae* under the control of the galactose-inducible GAL promoter in the presence of exogenously supplied substrate α-linolenic acid (ALA) and galactose (**C**). The presence of the Δ6-desaturation product stearidonic acid (SDA) is indicated (arrowed). In the absence of galactose no conversion of ALA is seen (**B**). The profile of control yeast strain transformed with empty vector is also shown (**A**). The substrate supplemented to the cultures is indicated by an asterisk.

The substrate specifiity of the putative Δ6-desaturase was determined by exogenously supplying various substrate fatty acids in the growth media. As shown in Figure 3, heterologous expression of the codon-optimized synthetic ORF encoding *Ostreococcus* RCC809 Protein 59992, predicted to encode a C18 Δ6-desaturase of 461 amino acids (aa), confirmed the enzymatic capability to convert exogenously supplied substrate α-linolenic acid (ALA; *n*-3) to the Δ6-desaturated product stearidonic acid (SDA; 18:4*n*-3). In the absence of galactose, the exogenous substrate ALA is not converted to SDA, since the transgene is not expressed. Thus, on the basis of these results, *Ostreococcus* RCC809 Protein 59992 was designated Ost809D6, and represents a new confirmed Δ6-desaturase member of the N-terminal cytochrome b5-fusion desaturase family. Ost809D6 displays high desaturation activity in yeast, converting about 54% of the available substrate ALA, with SDA accumulating to 18.5% of total fatty acids (Table 2). Ost809D6 only recognized the *n*-3 fatty acid ALA as a substrate, with the *n*-6 substrate LA showing no detectable desaturation in this heterologous yeast expression system. No activity was detected against exogenous 20:3*n*-6, 20:2*n*-6, 20:3*n*-3, 20:4*n*-3 and 22:5*n*-3. This strong preference for omega-3 *versus* omega-6 C18 substrates was also likely reflected in the fatty acid profile of *Ostreococcus* RCC809 (Supplementary Table S1), with accumulation of omega-6 (substrate) LA but not (product) GLA, and the inverse accumulation of omega-3 product (SDA) but not substrate (ALA). This strong omega-3 preference of Ost809D6 is distinct from a Δ6-desaturase with sequence-similarity identified from *Ostreococcus tauri* [14], which showed high activity towards both LA and ALA as substrates. It is more similar to Δ6-desaturase identified from *M. squamata* which accepts only ALA as a substrate [10]. Thus, Ost809D6 is potentially very useful for the exclusive production of Δ6-desaturated omega-3 fatty acids in transgenic plants.

**Table 2:** Fatty acid composition (%) of transgenic yeast. Values are the average of three independent experiments ± standard error. ND—not detected. The substrate supplemented to the culture is indicated in bold.

	Construct							
	Fatty Acid Composition (Molar %) ± SD							
Fatty Acid	Ost809D6 Gal −	Ost809D6 Gal +	Ost809D4 Gal −	Ost809D4 Gal +	FcElo6 Gal −	FcElo6 Gal +	FcElo6 Gal −	FcElo6 Gal +
**16:0**	22.3 ± 0.1	22.2 ± 0.4	32.8 ± 0.3	34.4 ± 0.2	24.5 ± 0.3	22.1 ± 0.1	20.1 ± 0.4	21.4 ± 0.1
**16:1**	25.4 ± 0.3	26.1 ± 0.3	38.9 ± 0.4	37.8 ± 0.3	26.8 ± 0.2	25.1 ± 0.3	22.3 ± 0.2	21.9 ± 0.3
**18:0**	3.7 ± 0.5	3.3 ± 0.3	7.9 ± 0.4	7.3 ± 0.5	3.7 ± 0.4	3.2 ± 0.3	3.7 ± 0.3	2.8 ± 0.4
**18:1**	13.4 ± 0.4	14.2 ± 0.4	17.8 ± 0.3	17.2 ± 0.4	15.3 ± 0.3	13.2 ± 0.5	3.4 ± 0.2	3.6 ± 0.3
**LA**	ND	ND	ND	ND	ND	ND	ND	ND
**GLA**	ND	ND	ND	ND	**29.7 ± 0.2**	**22.3 ± 0.1**	**28.2 ± 0.4**	**18.6 ± 0.5**
**ALA**	**32.9 ± 0.2**	**15.7 ± 0.1**	ND	ND	ND	ND	ND	ND
**SDA**	2.3 ± 0.6	18.5 ± 0.1	ND	ND	ND	ND	**22.3 ± 0.3**	**12.2 ± 0.4**
**20:4*n*-3**	ND	ND	ND	ND	ND	ND	ND	7.8 ± 0.3
**DGLA**	ND	ND	ND	ND	ND	14.1 ± 0.2	ND	11.7 ± 0.4
**DPA**	ND	ND	**2.6 ± 0.3**	**2.8 ± 0.2**	ND	ND	ND	ND
**DHA**	ND	ND	ND	0.5 ± 0.2	ND	ND	ND	ND

#### 2.1.3. Identification and Functional Characterization of a Putative Δ4-Desaturase from *Ostreococcus* RCC809

**Figure 4:**
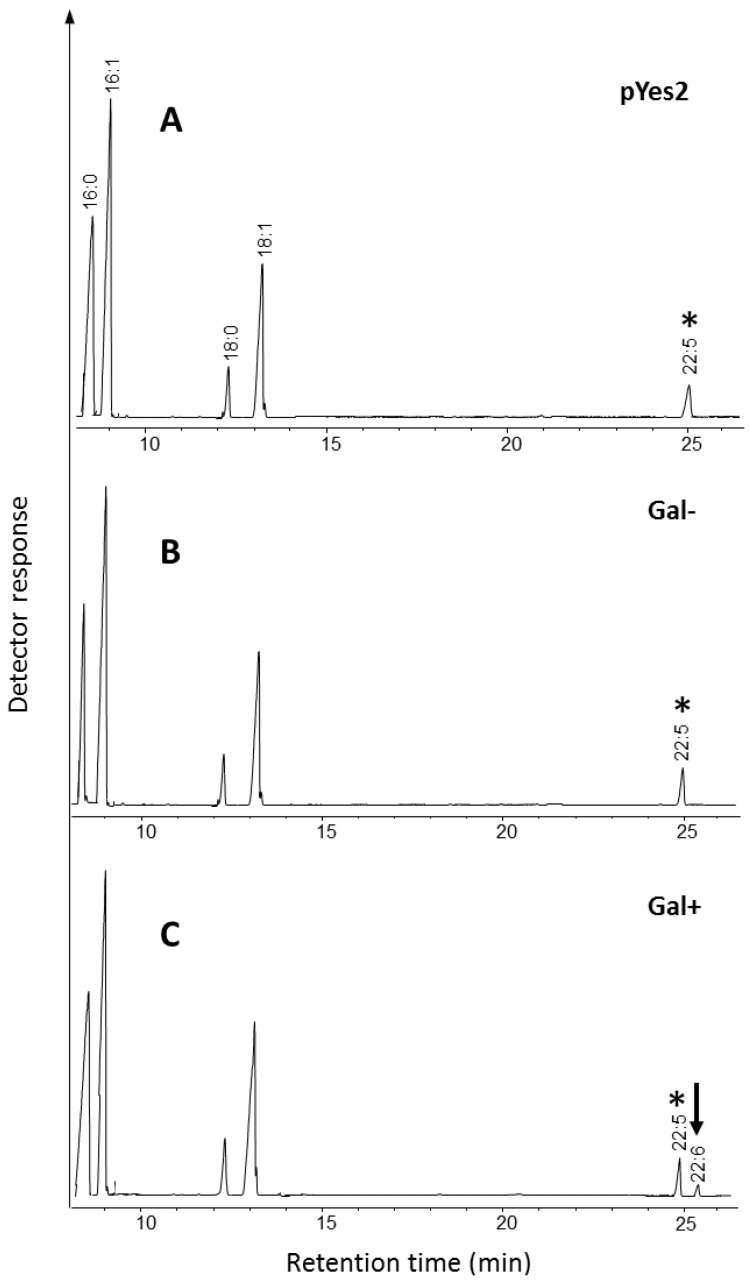
Functional characterization of *Ostreococcus* RCC809 C22 Δ4-desaturase. In the presence of exogenously supplied substrate (22:5*n*-3) and galactose, the accumulation of the Δ4-desaturation product DHA is detected (**C**) when total yeast fatty acids are analyzed. In the absence of galactose, no conversion of 22:5*n*-3 is seen (**B**). The profile of control yeast strain transformed with empty vector is also shown (**A**). The substrate supplemented to the cultures is indicated by an asterisk.

Similar to as described above, the genome sequence of *Ostreococcus* RCC809 was searched with previously functionally characterized sequences of Δ4-desaturases and the presence of an ortholog (JGI protein ID # 40461) for a Δ4-desaturase was detected (Table 1). The deduced amino acid sequence was used as a query for similarity searches using BLAST analyses after which a multiple alignment was created (Supplementary Figure S2). The most similar proteins were Δ4-desaturase from *O. lucimarinus* and chloroplast Δ6-desaturase from Chlamydomonas which had the identities of 85% and 41%, respectively, although the protein demonstrated very low similarity to previously reported Δ4-desaturases [12,15,19,20]. The deduced open reading frame was again used as a template to chemically synthesise codon-optimized nucleotide sequences for expression in the diatom *P. tricornutum*. The synthetic ORF of the putative Δ4-desaturase was inserted as a *KpnI-SacI* fragment behind the galactose-inducible GAL1 promoter of the yeast expression vector pYES2 and was tested for activity against the appropriate 22:5*n*-3 (DPA) substrate (Figure 4). Expression of the synthetic predicted ORF encoding a polypeptide of 459 aa resulted in the Δ4-desaturation of DPA to DHA, with a conversion rate of 15.1% (0.5% accumulation of DHA, Table 2), confirming the function of this ORF as a C22 Δ4-desaturase and on this basis we designated this gene as Ost809D4. Note that in the absence of the inducer (galactose), no DHA is detected, nor in the absence of the Ost809D4 ORF. No activity was detected against exogenously supplied potential substrate for Δ5-desaturation, 20:3*n*-6 (DGLA) (data not shown). Thus, although *Ostreococcus* RCC809 synthesizes only limited levels of DHA, its genome encodes a fully functional enzyme for the terminal desaturation in the biosynthesis of DHA. In that respect, it maybe be that under some particular environmental conditions or lifecycle stages this gene is more actively expressed and higher levels of DHA are generated.

### 2.2. Identification and Functional Characterization of a Putative Δ6-Elongase from *Fragilariopsis cylindrus*

#### 2.2.1. Fatty Acid Composition of *Fragilariopsis cylindrus*

**Figure 5:**
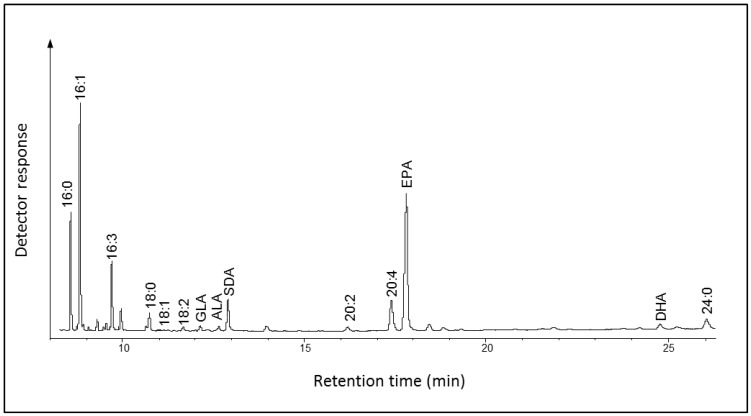
Total fatty acid methyl esters of *F. cylindrus*. FAMEs were prepared and analyzed by GC-FID as described in “Experimental Section”, with peaks being identified by co-migration against known standards. The identity of major peaks is shown.

The GC-FID analysis of FAMEs of total lipids from stationary phase cultures of *Fragilariopsis cylindrus* (*F. cylindrus*) (Figure 5) revealed that the most abundant fatty acid in this diatom was EPA (31.4% of TFA) followed by 16:1 and 16:0 (24.5% and 12.1% respectively) (Supplementary Table S1). Similar to *Ostreococcus* RCC809, only low levels of DHA were observed, although SDA levels were markedly lower in *F. cylindrus*. On the basis of significant levels of the C20 fatty acid EPA, it was indicative that this diatom contains a Δ6-elongase activity capable of elongating SDA to ETA prior to Δ5-desaturation, and for this reason, efforts were focused on identifying this gene.

#### 2.2.2. Functional Expression in Yeast of a Putative Elongase

**Figure 6:**
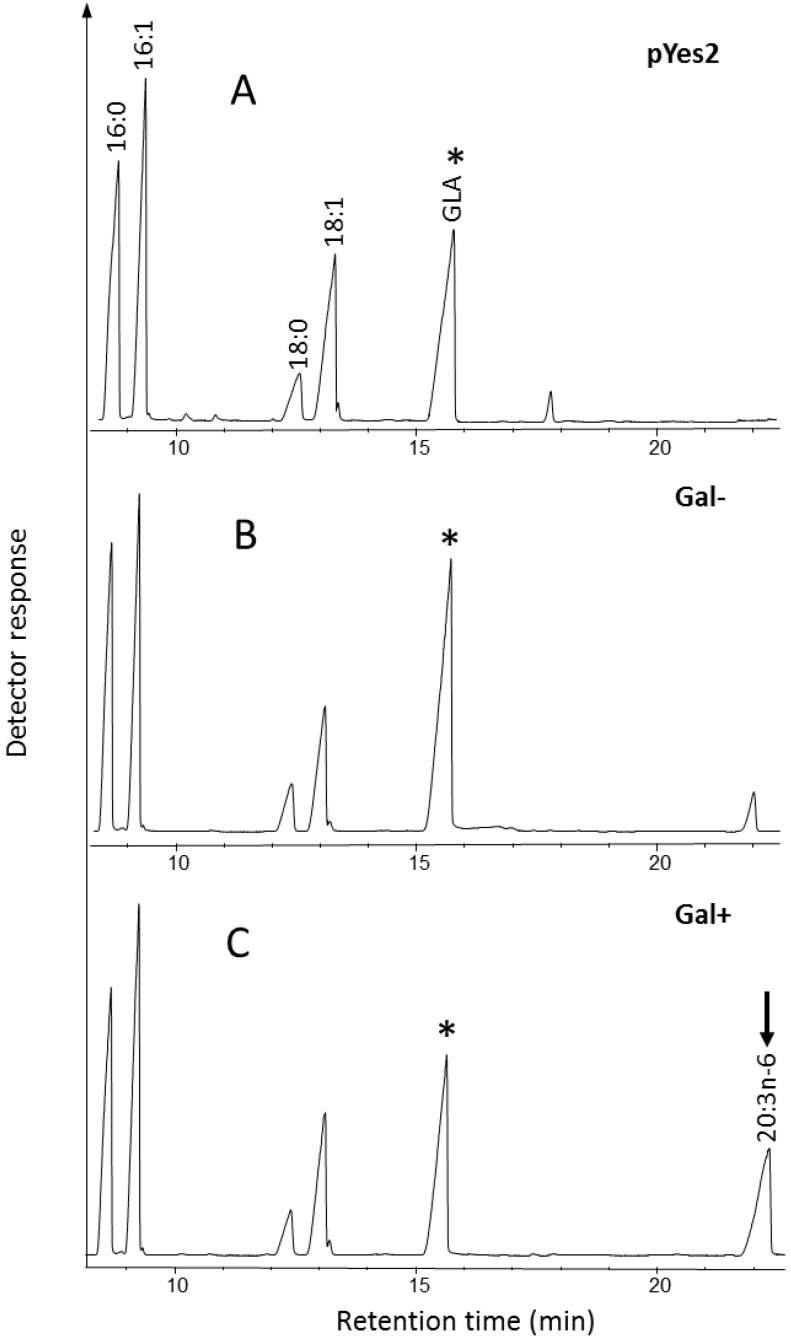
Functional characterization of *F. cylindrus* C18 Δ6-elongase. A synthetic gene encoding *F. cylindrus* Protein 177742 was expressed in *S. cerevisiae* under the control of the galactose-inducible GAL promoter. In the presence of exogenously supplied substrate (GLA) and galactose, the presence of the elongation product 20:3*n*-6 is detected when total yeast fatty acids are analyzed (**C**). In the absence of galactose, no elongation of GLA is seen (**B**). The profile of control yeast strain transformed with empty vector is also shown (**A**). The substrate supplemented to the cultures is indicated by an asterisk. Arrow indicates additional peak corresponding to DGLA, 20:3*n*-6.

Similar to the approach used for *Ostreococcus* RCC809, the publicly available genome sequence of the marine diatom *F. cylindrus* [21] was probed via BLAST search interface, using previously characterized Δ6-elongase sequences as the query template. This identified one strong candidate, and the deduced ORF of 287 aa (designated Frag#177742) was used as a query for similarity searches. Frag#177742 showed highest identity (70%) to Δ6-elongases from diatoms, *Thalassiosira pseudonana* [15] and *Phaeodactylum tricornutum* (Accession number AAW70157) (Supplementary Figure S3). To confirm the function of this putative elongase sequence, the synthetic ORF was expressed in yeast in the presence of exogenous 18:3*n*-6, GLA (Figure 6). Expression of Frag#177742 in yeast demonstrated that this sequence directed the elongation of GLA to generate 20:3*n*-6, DGLA (14.1% of TFA, 38.1% conversion rate). Thus, Frag#177742 was redesignated FcElo6, on the basis of possessing *bona fide* elongating activity specific for C18 Δ6-unsaturated substrates. The substrate specificity of FcElo6 was analyzed by exogenously supplying equal quantities of GLA and SDA in the growth media. As shown in Table 2, FcElo6 elongated SDA to generate 20:4*n*-3 at similarly high proportions (39% conversion rate). No elongation was observed with other exogenous potential substrates tested (LA and EPA, data not shown).

## 3. Experimental Section

### 3.1. Growth and Harvesting of Microalgal Strains

Cultures of *Ostreococcus* RCC809 were grown in ESAW medium [22,23] at 20 °C under 20 μM photons m^−2^ s^−1^. Cultures were agitated manually every two days to prevent aggregation. Cells were collected by centrifugation.

Cultures of *F. cylindrus* were grown in Aquil media [24,25,26] at 4 °C under illumination at 40 μM photons m^−2^ s^−1^, and were shaken manually from time to time. Cells were collected by centrifugation.

### 3.2. Identification and Cloning of Putative PUFA Genes

Genomes of the unicellular algae *Ostreococcus* RCC809 and the marine diatom *F. cylindrus* were analyzed with BLAST using *N*-terminal cytochrome b5-fusion desaturases and ELO-like elongating activity as templates. This analysis revealed the presence of several genes coding for putative PUFA desaturases and an elongase. The putative Δ6- and Δ4-desaturase sequences from *Ostreococcus* RCC809 and Δ6 elongase sequence from *F. cylindrus*, were used as templates to chemically synthesize (Genscript Corporation, Piscataway, NJ, USA) codon-optimized nucleotide sequences for expression in diatoms. The codon-optimized genes were subcloned into the *KpnI*-*SacI* sites present in the galactose inducible yeast expression vector pYES2 (Invitrogen, Carlsbad, CA, USA).

### 3.3. Functional Expression in Yeast

ORFs encoding putative desaturation and elongation activities were introduced in *S. cerevisiae* strain W303-1A by a lithium acetate method. Cultures were grown on synthetic dextrose minimal medium minus uracil at 22 °C in the presence of 2% (w/v) raffinose for 48 h and expression of the transgenes was induced by the addition of galactose to 2% (w/v) in the presence of 0.25 mM of the corresponding fatty acid and 1% (w/v) tergitol-Nonidet P-40 (Sigma-Aldricht, Haverhill, UK) as described [8].

### 3.4. Fatty Acid Analysis

Fatty acids were extracted and methylated as described previously [27]. Total fatty acids were analyzed by gas chromatography coupled with a flame ionization detector (GC-FID) of methyl ester derivatives. FAME samples were analyzed by as liquid chromatography using a Hewlett-Packard 6890 series Gas Chromatograph and an Alltech AT-225 (30 m × 0.32 mm × 0.3 µm) capillary column. Inlet and detector temperature was set to 250 °C and 1 µL of each sample was analyzed using splitless injection and a constant flow rate of 2 mL/min. The oven temperature cycle was set a follows: A start temperature of 50 °C was held for 1 min to allow vaporized samples and the solvent (hexane) to condensate at the front of the column. Oven temperature was then increased rapidly to 190 °C at a rate of 40 °C/min followed by a slower increase to 220° C at a rate of 1.5 °C/min. The final temperature of 220 °C was held for 1 min giving a total run time of 25 min 50 s per sample. FAMEs were detected using a Flame Ionization Detector (FID). Chromatograms were analysed using the Agilent ChemStation software Rev B.04.02 (118). Peak area percentages (area %) were converted to molecular percentages (mole %) to correct the error inherent to FID due to the different carbon number of each compound.

## 4. Conclusions

In conclusion, through a database search of the genomes of two primary producers of LC-PUFAs, we have identified and functionally characterized three novel genes involved in the biosynthesis of the nutritionally important omega-3 polyunsaturated fatty acids in marine microalgae. Of particular interest is the Δ6-desaturase from the unicellular photoautotrophic green alga *Ostreococcus* RCC809, which demonstrated activity with specificity towards omega-3 substrates, making this an interesting candidate for heterologous expression in transgenic plants. Previous studies by us [7,8] have identified unusual higher plant Δ6-desaturases from the *Primulaceae* which showed distinct preferences for either omega-3 or omega-6 C18 substrates. Similar to this present study, it was not possible to identify the precise amino acid determinants of such specificities, despite a high degree of similarity between enzymes with different substrate preferences. The *Ostreococcus* RCC809 activity described here is another example of an algal desaturase with strong selectivity for *n*-3 substrates and better conversion rates of the *n*-3 substarte ALA than for previously reported Δ6-desaturases from microalgae [10,11,12]. One additional factor which remains to be determined for this particular enzyme is the nature of substrate used by the desaturase. One of the most closely related orthologs of Ost809D6 is the Δ6-desaturase from *Ostreococcus tauri* (Supplementary Figure S2) which has been shown to prefer acyl-CoA substrates as opposed to the phospholipid-linked substrates more normally associated with lower eukaryotic desaturation [14]. Based on our present studies, it is not possible to infer any such tendency in the *Ostreococcus* RCC809 desaturase, but the definitive experiments remain to be carried out. Collectively, the algal genes functionally characterized in this study further add to our understanding of the biosynthesis of the vital and valuable omega-3 LC-PUFAs in the marine food-web, and also provide additional molecular tools with which to attempt the heterologous reconstruction of that biosynthetic pathway in transgenic hosts such as plants.

## Supplementary Files

Supplementary File 1 Supplementary Information (PDF, 623 KB)
