# Supplementary material for: Identification and Functional Characterization of Genes Encoding Omega-3 Polyunsaturated Fatty Acid Biosynthetic Activities from Unicellular Microalgae
Source: Mar Drugs. 2013 Dec 16;11(12):5116–29. doi: 10.3390/md11125116 (PMC3877907; doi:10.3390/md11125116)
Supplement: Supplementary File 1 — Supplementary Information (PDF, 623 KB) [file marinedrugs-11-05116-s001.pdf]

Supplementary Information

Table S1. Fatty acid composition of *Ostreococcus RCC809* (*O. RCC809*) and *F. cylindrus* cultures in stationary phase. Values are the average of three independent experiments (±standard deviation). ND—not detected.

Figure S1. Multiple sequence alignment of Ost809D6 with Δ6-desaturases from *Mantoniella squamata* (*M.squaD6,* CAQ30479.1) [1], *Micromonas pusilla* (*M.pusD6,* XP_002502445.1) [2], *Ostreococcus lucimarinus* (*O.lucD6,* DAA34893.1) [3] and *Ostreococcus tauri* (*O.tauriD6,* XP_003082578.1) [4]. Conserved histidine boxes are shaded in grey. Conserved amino acid residues are indicated with an asterisk. The position of conserved cytochrome b_5 domain motif is marked with a solid line. The alignment was obtained using CLUSTAL W.

Figure S2. Multiple sequence alignment of Ost809D4 with Δ4-desaturases from *Pavlova lutheri* (*P.lutD4*, AAQ98793.1) and *Pavlova salina* (*P.salD4*, AY926606.1) [4], *Isochrysis galbana* (*I.galD4*, AY630574) [5], *Ostreococcus lucimarinus* (*O.lucD4,* XM_001415706.1) [6], and *Emiliania huxleyi* (*E.huxD4*, [7]). Conserved histidine boxes are shaded in grey. Conserved amino acid residues are indicated with an asterisk. The position of conserved cytochrome b_5 domain motif is marked with a solid line. The alignment was obtained using CLUSTAL W. A related Δ4-desaturase from *Thalassiosira pseudonana* (TpD4, AAX14506.1) [8] was omitted from this line-up on the basis of poor alignment.

Figure S3. Multiple sequence alignment of FcElo6 with Δ6-elongasse from *Thalassiosira pseudonana* (TpElo6, AY591337.1) and *Ostreococcus tauri* (OtElo6, AY591335) [5]. Conserved amino acid residues are indicated with an asterisk, whereas conserved motifs are framed. The alignment was obtained using CLUSTAL W.

Figure S4. Codon-optimised nucleotide sequences. (**A**), Ost 809D6; (**B**), Ost809D4; (**C**) Fc ELO6.

Table S1. Fatty acid composition of *Ostreococcus RCC809* (*O. RCC809*) and *F. cylindrus* cultures in stationary phase. Values are the average of three independent experiments (±standard deviation). ND—not detected.

	**Composition (Molar %) ± SD**	
**Fatty Acid**	***O. RCC809***	***F. cylindrus***
**16:0**	20.12 ± 0.6	12.1 ± 0.2
**16:1*n*-7**	18.1 ± 0.3	24.5 ± 0.1
**16:4*n*-3**	5.08 ± 0.4	ND
**18:0**	1.24 ± 0.5	2.4 ± 0.3
**18:1*n*-9**	8.17 ± 0.2	ND
**18:1*n*-7**	8.03 ± 0.1	ND
**LA**	9.12 ± 0.2	0.7 ± 0.3
**GLA**	3.29 ± 0.3	1 ± 0.4
**ALA**	3.98 ± 0.4	0.8 ± 0.3
**SDA**	19.13 ± 0.6	5.7 ± 0.5
**20:2*n*-6**	0.65 ± 0.4	1 ± 0.2
**20:4*n*-3**	ND	7.4 ± 0.1
**EPA**	1.26 ± 0.4	31.4 ± 0.3
**DHA**	1.83 ± 0.5	2.5 ± 0.1
**24:0**	ND	1.4 ± 0.2

Figure S1. Multiple sequence alignment of Ost809D6 with Δ6-desaturases from *Mantoniella squamata* (*M.squaD6*, CAQ30479.1) [1], *Micromonas pusilla* (*M.pusD6*, XP_002502445.1) [2], *Ostreococcus lucimarinus* (*O.lucD6*, DAA34893.1) [3] and *Ostreococcus tauri* (*O.tauriD6*, XP_003082578.1) [4]. Conserved histidine boxes are shaded in grey. Conserved amino acid residues are indicated with an asterisk. The position of conserved cytochrome b_5 domain motif is marked with a solid line. The alignment was obtained using CLUSTAL W.

```
Ost809D6        -------------------MRVETEDDNVPTVTVGLSEESDGMKGARNPGARAWKSTLEP
O.lucD6         MCVETTEGTSRTMANERTSSSSSLSEGGTPTVTVGMGSE-DAGKKTRNASVTAWTKELEP
O.tauriD6       ------------------MCVETENNDGIPTVEIAFDGE----RERAEANVKLSAEKMEP
M.squaD6        ------------------------------MCPPKESTRKNAGGPLTRGKLSADLAKLEP
M.pusD6         -----------------------------------------MTRGNKAKLDNSKLAKLEP
                                                                         :**

Ost809D6        HAVAKSFDRRWVKVDGVEYDVTDFKHPGGSVIYYMLSNTGADATEAFKEFHYRSKKARKA
O.lucD6         HAIAKTFERRYVTIEGVEYDVTDFKHPGGSVIYYMLSNTGADATEAFKEFHYRSKKARKA
O.tauriD6       AALAKTFARRYVVIEGVEYDVTDFKHPGGTVIFYALSNTGADATEAFKEFHHRSRKARKA
M.squaD6        HKLAQTFDTRWVRVGDVEYDVTNFKHPGGSVIFYMLSNTGADATEAFNEFHMRSPKAWKM
M.pusD6         HKLAQTFEQRWVRIDDVEYDVTNFKHPGGSVIFYMLSNTGADATEAFKEFHMRSPKAWKM
                  :*::*  *:* : .******:******:**:* ************:*** ** ** *

Ost809D6        LAALPQREPEDAS--PVEDANMLKDFAKWRKDLEREGFFKPSPAHVAYRFAELAAMFALG
O.lucD6         LAALPHKPVDAATREPIEDEAMLKDFAQWRKELEREGFFKPSPAHVAYRFAELAAMFALG
O.tauriD6       LAALPSRP---AKTAKVDDAEMLQDFAKWRKELERDGFFKPSPAHVAYRFAELAAMYALG
M.squaD6        LKALPNRPAETPRSQ-DPDGPMLEDFAKWRAQLEKEGFFKPSIAHVAYRIAELAAMFALG
M.pusD6         LKALPQRPAETPRSA-DPDAPMLQDFARWRAELEKEGFFEPSRLHLAYRCLELCATFALG
                * *** :    .      *  **:***:** :**::***:**  *:***  **.* :***

Ost809D6        TALMYARWHATSVFVTACFFGARCGWVQHEGGHSSLTGSIWWDKRIQAFTAGFGLASSGD
O.lucD6         TALMHARWHVASVIVYSCFFGARCGWVQHEGGHNSLTGNIWWDKRIQAFAAGFGLASSGD
O.tauriD6       TYLMYARYVVSSVLVYACFFGARCGWVQHEGGHSSLTGNIWWDKRIQAFTAGFGLAGSGD
M.squaD6        CYIMSLGYPVVASIVFGAFFGARCGWVQHEGGHNSLTGNIWLDKRIQAATCGFGLSTSGD
M.pusD6         TFLMYIGRPLLASIVYGAFFGARCGWVQHEGGHNSLTGSIWWDKRIQAATCGFGLSTSGD
                  :*       : :* ..***************.****.** ****** :.****: ***

Ost809D6        MWNLMHNKHHATPQKVRHDMDLDTTPAVAFFNTAVEENRPRKFSKLWLRVQAWTFVPVTS
O.lucD6         MWNNMHNKHHATPQKVRHDMDLDTTPTVAFFNSAVEENRPRGFSKLWLRLQAWTFVPVTS
O.tauriD6       MWNSMHNKHHATPQKVRHDMDLDTTPAVAFFNTAVEDNRPRGFSKYWLRLQAWTFIPVTS
M.squaD6        MWNQMHNKHHATPQKVRHDMDLDTTPAVAFFKTAVEDNRPRGFSRAWSRAQAWTFVPVTS
M.pusD6         MWNQMHNKHHATPQKVRHDMDLDTTPAVAFFDTAVEDNRPRGFSKTWARAQAWTFVPITS
                *** **********************:****.:***:**** **: * * *****:*:**

Ost809D6        -GLVLLAWMYLLHPRHIARRKNYEEAAWIVAAHVIRTSVIKAVTGYSWITCYGLFLSTMW
O.lucD6         -GMVLFFWMFVLHPRNALRRKSFEEAAWMFSAHVIRTAVIKAVTGYSWIASYGLFAATMW
O.tauriD6       -GLVLLFWMFFLHPSKALKGGKYEELVWMLAAHVIRTWTIKAVTGFTAMQSYGLFLATSW
M.squaD6        GLLVQMFWIYVLHPRQVARKKNYEEASWMILSHVLRTATIKYAGGYSWPVAYLWFSFGNW
M.pusD6         GVLVQMFWIYVLHPRQVLRKKNYEEASWMFLSHVVRTAVIKLALGCGTAEAYGWFWVGNW
                  :* : *::.*** :  :  .:**  *:. :**:** .** . *     .*  *    *

Ost809D6        VSGCYLFAHFSTSHTHLDVVPSDKHLSWVRYAVDHTIDIDPSKSVVNWLMGYLNCQVIHH
O.lucD6         ASGCYLFAHFSTSHTHLDVVPSDKHLSWVRYAVDHTIDINPNNSVVNWLMGYLNCQVIHH
O.tauriD6       VSGCYLFAHFSTSHTHLDVVPADEHLSWVRYAVDHTIDIDPSQGWVNWLMGYLNCQVIHH
M.squaD6        IAYMYLFAHFSTSHTHLEVVPSDKHISWVNYAVDHTVDIDPSKGYVNWLMGYLNCQVIHH
M.pusD6         IAYMYLFAHFSTSHTHLDVVPSDKHISWVNYAVDHTVDINPRNSIVNWLMGYLNCQVIHH
                 :  *************:***:*:*:***.******:**:* :. ***************

Ost809D6        LFPDMPQFRQPEVSRRFVSFAKKWNLNYKVMSYYGAWKATFGNLNEVGKHYYIQGSQITK
O.lucD6         LFPDMPQFRQPEVSRRFVPFAKKWNLNYKVLTYYGAWKATFGNLNDVGKHYYVHGSQRVK
O.tauriD6       LFPSMPQFRQPEVSRRFVAFAKKWNLNYKVMTYAGAWKATLGNLDNVGKHYYVHGQHSGK
M.squaD6        LFPDMPQFRQPEVSRRFVAFAKKWNLNYKVLTYYGAWKATFTNLDTVGQHYYKHGKAHAH
M.pusD6         LFPDMPQFRQPEVSRRFVAFAEKWNLNYKVLTYYGAWKATFSNLDRVGQHYYVNGKAKAH
                ***.**************.**:********::* ******: **: **:*** :*.   :
```

Figure S2. Multiple sequence alignment of Ost809D4 with Δ4-desaturases from *Pavlova lutheri* (*P.lutD4*, AAQ98793.1) and *Pavlova salina* (*P.salD4*, AY926606.1) [4], *Isochrysis galbana* (*I.galD4*, AY630574) [5], *Ostreococcus lucimarinus* (*O.lucD4*, XM_001415706.1) [6] and *Emiliania huxleyi* (*E.huxD4*, [7]). Conserved histidine boxes are shaded in grey. Conserved amino acid residues are indicated with an asterisk. The position of conserved cytochrome b_5 domain motif is marked with a solid line. The alignment was obtained using CLUSTAL W. A related Δ4-desaturase from *Thalassiosira pseudonana* (TpD4, AAX14506.1) [8] was omitted from this line-up on the basis of poor alignment.

```
Ost809D4        ---MPTTRSRARVTTPPRETPTRANTVAALDPERKYTRIRGVVYDVTDFASRHPGGAQLL
O.lucD4         --MPSAARSRASKRANATTDVATTAPEATLDPTRAYTRYRGVVYDVTEFQHRHPGGAQLL
P.lutD4         MPPSAASEGGVAELRAAEVAS---YTRKAVDERPDLTIVGDAVYDAKAFRDEHPGGAHFV
P.salD4         MPPSAAKQMGASTGVHAGVTDSSAFTRKDVADRPDLTIVGDSVYDAKAFRSEHPGGAHFV
I.galD4         --------------MCNAAQVETQALRAKEAAKPTWTKIHGRTVDVETFR--HPGGN-IL
EhuxD4          ----------------------MGGAGASEAERPKWTTIHGRHVDVSKFR--HPGGN-II
                                                    *   .   *.  *   ****  ::

Ost809D4        SLCVGRDATILVESHHLRPEVVQKYLKTLPVVEGAAGAFGPEETFPKPLDSDLYRKIQGR
O.lucD4         SLCVGRDATILIESHHLRPEVVRKYMKTLPVVEGAAGAFGKEETFPKPLDSAVYRAIQRR
P.lutD4         SLFGGRDATEAFMEYHRRAWPKARMSKFFVGSLDASEKPTQADSAYLRLCAEVNALLPKG
P.salD4         SLFGGRDATEAFMEYHRRAWPKSRMSRFHVGSLASTEEPVAADEGYLQLCARIAKMVPSV
I.galD4         DLFLGMDATTAFETFHGHHKGAWKMLKTLPEKEVAAADIPAQKEEHVAEMTRLMASWRER
EhuxD4          ELFYGMDSTSAFEQFHGHHKGAWKMLKALPTKEVDPADVPQQPQEHVAEMTRLMTSWRER
                .*  * *:*  .  .* :     :  :        .              : :

Ost809D4        VRKEIVEPLKMTRGREPHGRGWCVLDAGVVLAFFAFALGVYWKTPTVATGCLLGLAGYWS
O.lucD4         VRDEVVEPMKAKSGREAHGRGGCVVDAGVVVLTFVAAMVAYWRAPSALTGCALGLAGYWS
P.lutD4         SGGFAPPSYWLKAA-------------ALVVAAVSIEGYMLLRGKTLLLSVFLGLVFAWI
P.salD4         SSGFAPASYWVKAG-------------LILGSAIALEAYMLYAGKRLLPSIVLGWLFALI
I.galD4         GLFKPRPVASSIYG--------------LCVIFAIAASVACAPYAPVLAGIAVGTCWAQC
EhuxD4          GLFKPRPVASGIYG--------------LAVVAAIVACIACAPHAPVLSGIGLGSCWAQC
                             .              :                    .  :*

Ost809D4        GTGLQHTANHGGLAKSGFWNQFWGWLGNDVAIGKSSVEWRYHHMVSHHSYCNDADLDQDV
O.lucD4         GTGLQHTANHGGLAKSGFWNQFWGWLGNDVAIGKSSVEWRYHHMVSHHSYCNDADLDQDV
P.lutD4         GLNIQHDANHGALSRHSVINYCLGYA--QDWIGGNMVLWLQEHVVMHHLHTNDVDADPDQ
P.salD4         GLNIQHDANHGALSKSASVNLALGLC--QDWIGGSMILWLQEHVVMHHLHTNDVDKDPDQ
I.galD4         G-FLQHMGGHREWGRTWSFAFQHLFEG---LLKGGSASWWRNRHNKHHAKTNVLGEDGD-
EhuxD4          G-FLQHMGGHREWGVRYSFLLQHFFEG---LLKGGSASWWRNRHNKHHAKTNVLGEDGD-
                *  :** ..*   .                 :  .   *  .:   **   *  . * *

Ost809D4        YTALPLLRLDPSQELKWFHRYQAFYAPLMWPMLWLAAQFGDAQNILVDKASPGVEYKGLM
O.lucD4         YTALPLLRLDPSQELKWFHRYQAFYAPLMWPFLWLAAQVGDAQNILIDRASPGVEYKGLM
P.lutD4         K-AHGVLRLKPTDGWMPWHALQQLYILPGEAMYAFKLLFLDALELLAWRWE-GEKISPLA
P.salD4         K-AHGALRLKPTDAWSPMHWLQHLYLLPGETMYAFKLLFLDISELVMWRWE-GEPISKLA
I.galD4         ------LRTTPFFAWDP----TLAKKVPDWSLRTQAFTFLPALGAYVFVFAFTVRKYSVV
EhuxD4          ------LRTTPFFAWDP----TLAKKVPDWSLKTQAFTFLPALGAYVFVFAFTIRKYAVV
                      **  *                   .:      .                   :

Ost809D4        KLEVALYVLGKFLHFSLLLGVPAYLHGFANAIVPFIAYGAFGSFVLCWFFIVSHNLEALT
O.lucD4         KNEIALYLLGKVLHFGLLLGVPAYIHGLSNVIVPFLAYGAFGSFVLCWFFIVSHNLEALT
P.lutD4         RALFAPAVACKLGFWARFVALPLWLQPTVHTALCICATVCTGSFYLAFFFFISHNFDGVG
P.salD4         GYLFMPSLLLKLTFWARFVALPLYLAPSVHTAVCIAATVMTGSFYLAFFFFISHNFEGVA
I.galD4         KRLWHEVALMVAHYALFSWALSAAGASLSSGLTFYCTGYAWQGIYLGFFFGLSHFAVERV
EhuxD4          KKLWHELALMIAHYAMFYYALQLAGASLGSGLAFYCTGYAWQGIYLGFFFGLSHFAVERV
                             .     .:               :     .: * :** :**

Ost809D4        PIN-----LSKSTKNDWGAWQIETSASWGN--GFWSFFSGGLNLQIEHHLFPGCAHNLYP
O.lucD4         PMN-----LSKSTKNDWGAWQIETSASWGN--SFWSFFSGGLNLQIEHHLFPGCAHNLYP
P.lutD4         SVGPKG---SLPRSATFVQRQVETSSNVGG--YWLGVLNGGLNFQIEHHLFPRLHHSYYA
P.salD4         SVGPDGSITSMTRGASFLKRQAETSSNVGG--PLLATLNGGLNYQIEHHLFPRVHHGFYP
I.galD4         PS-----------TATWLESTMMGTVDWGGSSAFCGYLSGFLNIQIEHHMAPQMPMENLR
EhuxD4          PS-----------TATWLESSMIGTVDWGGSSAFCGYVSGFLNIQIEHHMAPQMPMENLR
                .               :       : . *.     . ..* ** *****: *
```

Figure S3. Multiple sequence alignment of FcElo6 with Δ6-elongasse from *Thalassiosira pseudonana* (TpElo6, AY591337.1) and *Ostreococcus tauri* (OtElo6, AY591335) [5]. Conserved amino acid residues are indicated with an asterisk, whereas conserved motifs are framed. The alignment was obtained using CLUSTAL W.

```
FcElo6          ---------------------MDEYKATLESVGDAIIQWADPESQFTGFTKGWFLTDFTS
TpElo6          ---------------------MDAYNAAMDKIGAAIIDWSDPDGKFRADREDWWLCDFRS
OtElo6          MSGLRAPNFLHRFWTKWDYAISKVVFTCADSFQWDIGPVSSSTAHLPAIESPTPLVTSLL
                                      .   :  :..   *   :.. .:: .  .   *

FcElo6          AFSIALVYVLFVIIGSQVMKVLPAIDPYPIKFFYNVSQIMLCAYMTIEACLLAYRNGYTI
TpElo6          AITIALIYIAFVILGSAVMQSLPAMDPYPIKFLYNVSQIFLCAYMTVEAGFLAYRNGYTV
OtElo6          FYLVTVFLWYGRLTRSSDKKIREPTWLRRFIICHNAFLIVLSLYMCLGCVAQAYQNGYTL
                   :::.     :  *   :   .     : : :*.  *.*. ** : .   **:****:

FcElo6          MPCVGYNRDDPAIGNLLWLFYVSKVWDFWDTIFIVLGKKWRQLSFLHVYHHTTIFLFYWL
TpElo6          MPCNHFNVNDPPVANLLWLFYISKVWDFWDTIFIVLGKKWRQLSFLHVYHHTTIFLFYWL
OtElo6          WG-NEFKATETQLALYIYIFYVSKIYEFVDTYIMLLKNNLRQVSFLHIYHHSTISFIWWI
                     ::  :. :.  :::**:**:::* ** :::* :: **:****:***:** :::*:

FcElo6          NANVFYDGDIYLTIALNGFIHTVMYTYYFICMHTKDKKTGKSLPIWWKSSLTLLQLFQFI
TpElo6          NANVLYDGDIFLTILLNGFIHTVMYTYYFICMHTKDSKTGKSLPIWWKSSLTAFQLLQFT
OtElo6          IARRAPGGDAYFSAALNSWVHVCMYTYYLLSTLIGKEDPKRSNYLWWGRHLTQMQMLQFF
                 *.   .** :::  **.::*. *****::.    .... :*  :**   ** :*::**

FcElo6          TMMSQGLYLIIFGCESLSIRVTATYVVYILSLFFLFAQFFVASYMQPKKSKTA-ELGTLI
TpElo6          IMMSQATYLVFHGCDKVSLRITIVYFVSLLSLFFLFAQFFVQSYMAPKKKKSA-------
OtElo6          FNVLQALYCASF--STYPKFLSKILLVYMMSLLGLFGHFYYSKHIAAAKLQKKQQ-----
                  : *. *    .  .. .  ::   .* ::**: **.:*:  .:: . * :.
```

Figure S4. Codon-optimised nucleotide sequences. (**A**), Ost 809D6; (**B**), Ost809D4; (**C**) Fc ELO6.

A

1 ATGCGTGTGGAAACCGAAGACGATAATGTGCCAACTGTTACTGTGGGATTGTCAGAGGAG

61 TCCGATGGAATGAAGGGAGCAAGGAACCCCGGAGCACGTGCTTGGAAGTCGACGTTGGAG

121 CCGCACGCCGTGGCAAAGTCATTCGATCGTAGGTGGGTTAAGGTTGACGGAGTCGAATAC

181 GACGTAACTGATTTCAAGCATCCCGGAGGATCAGTTATCTACTATATGCTTTCTAACACC

241 GGAGCTGATGCCACTGAGGCTTTCAAGGAATTTCACTATCGTAGTAAGAAGGCCAGGAAG

301 GCACTTGCTGCCCTCCCACAACGTGAGCCTGAAGACGCTTCGCCAGTCGAGGATGCCAAT

361 ATGCTCAAGGACTTCGCAAAGTGGCGTAAGGATTTGGAGAGGGAAGGATTCTTTAAGCCA

421 AGTCCTGCTCACGTGGCCTACCGTTTCGCCGAACTCGCAGCTATGTTTGCTTTGGGAACT

481 GCCCTTATGTATGCACGTTGGCATGCTACGTCTGTCTTCGTAACAGCCTGTTTCTTTGGA

541 GCAAGGTGTGGATGGGTGCAACACGAGGGAGGACATTCTTCCTTGACCGGATCCATCTGG

601 TGGGATAAGCGTATTCAGGCATTCACTGCTGGATTTGGACTTGCCAGTTCGGGAGACATG

661 TGGAACCTCATGCACAATAAGCACCATGCAACGCCACAAAAAGTTAGGCATGATATGGAC

721 CTCGATACCACTCCTGCAGTGGCTTTCTTTAACACAGCTGTTGAGGAAAATCGTCCTAGG

781 AAGTTCTCTAAGTTGTGGCTTCGTGTCCAGGCCTGGACCTTTGTGCCCGTTACTTCCGGA

841 TTGGTACTCTTGGCATGGATGTACCTTCTCCACCCGCGTCATATCGCTCGTAGGAAGAAC

901 TATGAGGAAGCCGCATGGATTGTGGCTGCCCATGTTATCAGGACCTCCGTCATTAAGGCT

961 GTAACGGGATACAGTTGGATCACATGTTATGGACTCTTCTTGTCGACTATGTGGGTCTCA

1021 GGATGCTACCTCTTCGCTCACTTTTCAACGTCTCACACACATTTGGACGTGGTTCCATCT

1081 GATAAGCACCTTTCCTGGGTGCGTTACGCCGTTGATCATACCATCGACATTGATCCTTCC

1141 AAGAGTGTCGTAAACTGGCTCATGGGATATTTGAACTGTCAGGTTATCCACCATTTGTTC

1201 CCCGACATGCCGCAATTTCGTCAGCCCGAAGTCAGTCGTAGGTTCGTATCGTTTGCCAAG

1261 AAGTGGAACCTTAATTACAAGGTCATGTCTTACTATGGAGCCTGGAAGGCAACCTTCGGA

1321 AATCTCAACGAAGTCGGAAAGCACTACTACATCCAAGGAAGTCAAATCACAAAGAAGACG

1381 GTTTAG

Figure S4. *Cont.*

B

1 ATGCCAACTACTCGTTCTCGTGCTCGTGTTACTACTCCACCTCGTGAAACTCCTACTCGT

61 GCTAATACTGTTGCTGCTTTAGATCCAGAACGTAAATATACACGTATTCGAGGTGTTGTA

121 TATGATGTTACTGATTTTGCTAGTCGACATCCAGGTGGTGCACAATTATTATCTTTATGT

181 GTTGGTCGTGATGCTACAATTTTAGTAGAATCACATCATTTACGACCAGAAGTTGTACAA

241 AAATATTTAAAAACATTACCTGTTGTAGAAGGTGCTGCTGGTGCATTTGGTCCAGAAGAA

301 ACTTTTCCAAAACCTTTAGATAGTGATTTATATCGTAAAATTCAAGGTCGTGTTCGAAAA

361 GAAATTGTAGAACCATTAAAAATGACACGTGGTCGAGAACCTCATGGTCGTGGTTGGTGT

421 GTTTTAGATGCTGGTGTTGTATTAGCTTTCTTTGCTTTTGCATTAGGTGTTTATTGGAAA

481 ACACCAACTGTAGCTACTGGTTGTTTATTAGGTTTAGCAGGTTATTGGTCTGGTACAGGT

541 TTACAACATACTGCTAATCATGGTGGTTTAGCAAAATCAGGTTTTTGGAATCAATTTTGG

601 GGTTGGTTAGGAAATGATGTTGCTATTGGTAAATCAAGTGTAGAATGGCGTTATCATCAT

661 ATGGTTTCACATCATAGTTATTGTAATGATGCTGATTTAGATCAAGATGTTTATACAGCA

721 TTACCATTATTACGTTTAGATCCTTCACAAGAATTAAAATGGTTTCATCGTTATCAAGCA

781 TTTTATGCACCTTTAATGTGGCCTATGTTATGGTTAGCTGCACAATTTGGTGATGCTCAA

841 AATATTTTAGTTGATAAAGCAAGTCCAGGTGTAGAATATAAAGGTTTAATGAAATTAGAA

901 GTTGCTTTATATGTATTAGGAAAATTTTTACATTTTTCTTTATTATTAGGTGTTCCTGCA

961 TATTTACATGGTTTTGCTAATGCAATTGTACCATTTATTGCTTATGGTGCATTTGGTTCA

1021 TTTGTTTTATGTTGGTTTTTCATTGTAAGTCATAATTTAGAAGCATTAACACCAATTAAT

1081 TTATCTAAATCAACTAAAAATGATTGGGGTGCTTGGCAAATTGAAACTAGTGCATCTTGG

1141 GGTAATGGTTTTTGGTCATTTTTCTCAGGTGGTTTAAATTTACAAATTGAACATCATTTA

1201 TTTCCTGGTTGTGCTCATAATTTATATCCAAAAATGGTTCCTATTATTAAAGAAGAATGT

1261 GAAAAAGCAGGTGTTACATATACTGGTTATGGTGGTTATTTTGGTTTATTACCAATTACT

1321 CGTGATATGTTTGCTTATTTATATAAAATGGGTCGTCAATCTAAAAAATCTGCTTAA

Figure S4. *Cont.*

C

```
1 ATGGATGAGTACAAGGCCACTTTGGAGTCAGTAGGAGACGCTATTATTCAATGGGCAGAC

61 CCCGAGTCACAGTTCACCGGATTCACTAAGGGATGGTTCCTCACCGATTTTACTTCAGCT

121 TTCTCTATCGCCCTCGTCTACGTATTGTTCGTGATCATTGGATCCCAAGTGATGAAGGTT

181 CTCCCTGCTATCGACCCATACCCTATCAAGTTCTTTTATAACGTTAGTCAGATCATGTTG

241 TGTGCCTATATGACCATTGAGGCATGCCTCTTGGCTTACCGTAATGGATATACTATCATG

301 CCCTGTGTCGGATACAACAGGGATGACCCGGCAATTGGAAATCTTCTCTGGCTTTTCTAT

361 GTCTCGAAAGTATGGGATTTTTGGGACACGATCTTCATTGTGTTGGGAAAGAAGTGGCGT

421 CAATTGTCATTTCTTCACGTTTACCACCATACCACTATCTTCCTCTTCTACTGGCTCAAC

481 GCCAATGTCTTCTACGATGGAGACATCTATTTGACCATTGCACTTAACGGATTTATCCAC

541 ACGGTAATGTACACATACTACTTCATCTGTATGCATACCAAGGATAAGAAGACTGGAAAG

601 AGTCTCCCAATCTGGTGGAAGTCTTCCCTCACGTTGCTTCAATTGTTCCAGTTCATCACA

661 ATGATGTCTCAGGGACTCTACTTGATCATTTTCGGATGCGAAAGTTTGTCGATCAGGGTG

721 ACGGCCACATACGTGGTTTATATTCTTTCTCTCTTCTTTTTGTTCGCCCAGTTTTTCGTC

781 GCCTCCTACATGCAGCCCAAGAAGTCCAAGACAGCCTGA
```

References

1. Hoffmann, M.; Wagner, M.; Abbadi, A.; Fulda, M.; Feussner, I. Metabolic engineering of omega3-very long chain polyunsaturated fatty acid production by an exclusively acyl-CoA-dependent pathway. *J. Biol. Chem*. **2008**, *283*, 22352–22362.
2. Petrie, J.R.; Shrestha, P.; Mansour, M.P.; Nichols, P.D.; Liu, Q.; Singh, S.P. Metabolic engineering of omega-3 long-chain polyunsaturated fatty acids in plants using an acyl-CoA Delta6-desaturase with omega-3 preference from the marine microalga *Micromonas pusilla*. *Metab. Eng.* **2010**, *12*, 233–240.
3. Petrie, J.R.; Liu, Q.; Mackenzie, A.; Shrestha, P.; Mansour, M.P.; Robert, S.S.; Frampton, D.F.; Blackburn, S.I.; Nichols, P.D.; Singh, S.P. Isolation and characterization of a high-efficiency desaturase and elongases from microalgae for transgenic LC-PUFA production. *Mar. Biotechnol*. **2010**, *12*, 430–438.
4. Domergue, F.; Abbadi, A.; Zähringer, U.; Moreau, H.; Heinz, E. *In vivo* characterization of the first acyl-CoA Delta6-desaturase from a member of the plant kingdom, the microalga *Ostreococcus tauri*. *Biochem. J.* **2005**, *389*, 483–490.
5. Meyer, A.; Kirsch, H.; Domergue, F.; Abbadi, A.; Sperling, P.; Bauer, J.; Cirpus, P.; Zank, T.K.; Moreau, H.; Roscoe, T.J.; *et al.* Novel fatty acid elongases and their use for the reconstitution of docosahexaenoic acid biosynthesis. *J. Lipid Res*. **2004**, *45*, 1899–1909.
6. Ahman, K.; Heilmann, M.; Feussner, I. Identification of a Δ4-desaturase from microalga *Ostreococcus lucimarinus*. *Eur. J. Lipid Sci. Technol*. **2011**, *113*, 832–840.

7. Sayanova, O.; Haslam, R.; Venegas-Calerón, M.; Ruiz-Lopez, N.; Worthy, C.; Rooks, P.; Allen, M.J.; Napier, J.A. Identification and functional characterization of genes encoding the omega-3 polyunsaturated fatty acid biosynthetic pathway from the coccolithophore *Emiliania huxleyi*. *Phytochemistry* **2011**, *72*, 594–600.
8. Tonon, T.; Sayanova, O.; Michaelson, L.V.; Qing, R.; Harvey, D.; Larson, T.R.; Li, Y.; Napier, J.A.; Graham, I.A. Fatty acid desaturases from the microalga *Thalassiosira pseudonana*. *FEBS J.* **2005**, *272*, 3401–3412.

© 2013 by the authors; licensee MDPI, Basel, Switzerland. This article is an open access article distributed under the terms and conditions of the Creative Commons Attribution license (http://creativecommons.org/licenses/by/3.0/).
